# The plastid genome of Vanillon (*Vanilla pompona*, Orchidaceae)

**DOI:** 10.1080/23802359.2017.1383201

**Published:** 2017-09-29

**Authors:** Ali Amiryousefi, Jaakko Hyvönen, Péter Poczai

**Affiliations:** a Finnish Museum of Natural History (Botany), University of Helsinki, Helsinki, Finland;; b Department of Bioscience (Plant Biology), Viikki Plant Science Centre, University of Helsinki, Helsinki, Finland

**Keywords:** Chloroplast genome, *de novo* assembly, genome skimming, phylogenomics, plastid evolution

## Abstract

This study presents the complete sequence of *Vanilla pompona* chloroplast genome. This 148,009 bp long genome consist of 107 genes out of which 30 of them are tRNA, 4 rRNA. Fairly long inverted repeat regions (IR) and large single-copy (LSC) of length 29,807 and 86,358 bp, respectively, were detected. This means an exceptionally short single-copy (SSC) region with only 2037 bp. This truncation of the SSC is due to multiple translocation of *ndh* genes to the mitochondrion as in majority of the Orchidaceae and especially in the genus Vanilla. The phylogeny presented here meaningfully places Vanillon within the orchid family.

Vanilla is the world’s most popular flavour and, by unit mass, is among the most valuable of the spice crops (Bory et al. 2008). The main source of vanilla aroma is *Vanilla planifolia* Andrews, and it is the second most expensive spice next only to saffron (Ranadive 1994). The world production of vanilla was estimated to be about 8032 tons in 2014 (FAOSTAT 2017). Two other species *V. tahitensis* J.W.Moore and *V. pompona* Schiede are also cultivated. *Vanilla pompona* is extremely rare, grown only in Guadeloupe, Martinique and Dominica and used only by the cosmetic and perfume industries due to its floral/perfumery/heliotropin-like flavour (Ehlers and Pfister 1997). Besides being economically important, Orchidaceae are an appealing family for evolutionary studies due to multiple translocation of the *ndh* genes from the chloroplast to the mitochondrion (Lin et al. 2015). The loss or dysfunctioning of these genes in the plastome seems especially bold in Vanilloideae since their plastid genome exhibit only one functional *ndh* gene.

We extracted DNA according to Shi et al. (2012) from 20 g fresh Vanillon leaves collected in Kaisaniemi Botanical Garden, University of Helsinki, Finland (60.1753; 24.9460; voucher M. Christenhusz 00YY-845). Paired-end libraries of 2 × 150 bp were prepared with Illumina TruSeq DNA Sample prep kit and sequencing was carried out on an Illumina MiSeq platform. Raw reads were filtered with Trimmomatic (Bolger et al. 2014), and *de novo* assembly of the plastid genome was carried out with the Geneious assembler platform (Kearse et al. 2012). We annotated the genome using DOGMA (Wyman et al. 2004) following manual correction in Geneious. Here, we report the complete chloroplast sequence of *Vanilla pompona* to provide resources for taxonomic studies and plant breeding.

The complete chloroplast genome of *Vanilla pompona* (GenBank accession MF197310) has a total length of 148,009 bp which is divided by two IR regions of 29,807 bp. This genome comprises of 107 genes and has 34% overall GC content. The genes are classified into 30 tRNA, 4 rRNA and 75 coding-protein genes. From the 11 *ndh* plastome encoded NAD(P)H dehydrogenase complex genes (Ueda et al. 2012), only *ndh*B was detected in the plastid genome of *V. pompona*. This indicates the loss or pseudogenization of the rest. This independent and complex pattern of loss of functional *ndh* genes in Orchidaceae is not tied with a significant evolutionary events (Kim et al. 2015), but justifies the exceptionally short length of SSC in the Vanilloideae.

Using the RAxMLv8.0 (Stamatakis 2014) the best scoring ML tree with 10,000 bootstrap replicates was calculated under GTR-GAMMA after running jModelTest2 (Darriba et al. 2012) including 18 representative species in Orchidaceae (Figure 1). As expected *Vanilla pompona* was resolved as sister to *V. planifolia* and together they formed a basal clade to Cypripedioideae, Orchidoideae, and Epidendroideae. We also made phylogenetic analysis using parsimony as an optimality criterion. The same matrix as with ML was analyzed using Nona (Goloboff 1994) within the WinClada shell (Nixon 2002).

**Figure 1.**
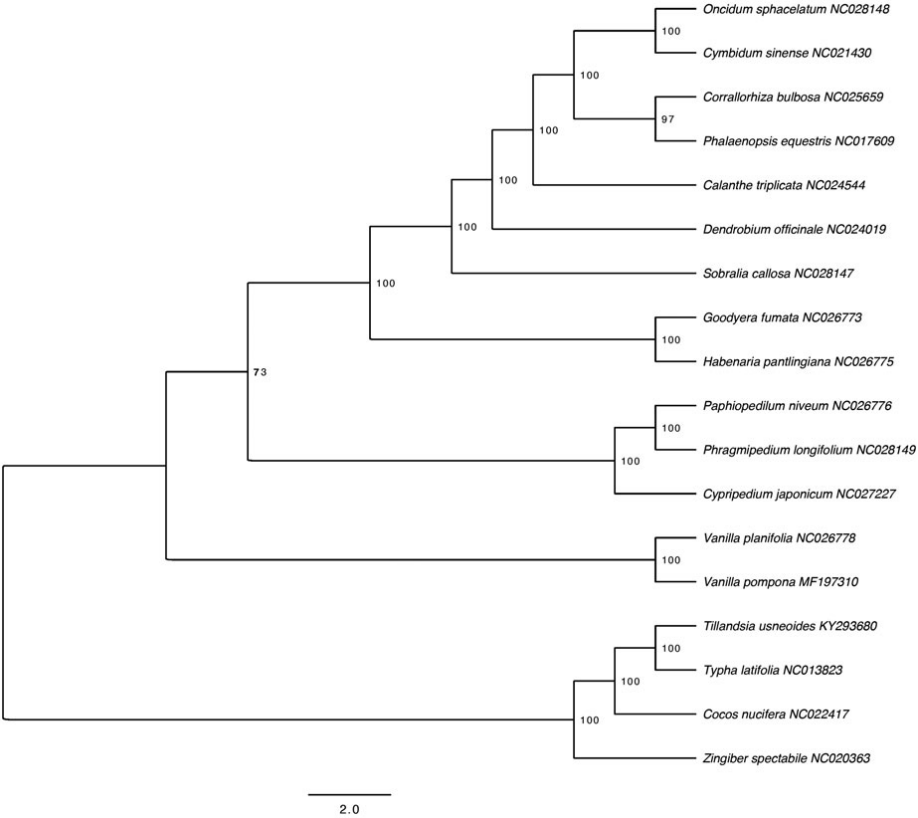
The ML tree of 17 selected Orchidaceae chloroplast sequences plus *Vanilla pompona*. The values on the node show the bootstraps of 10,000 replicates and scale is substitution per site. New paragraph: use this style when you need to begin a new paragraph.

We expect this sequence to clarify the taxonomic status of *Vanilla pompona*, and provide additional genomic resources for vanilla breeding.
